# Blocksom-Type Cutaneous Vesicostomy in Adult Patients With Severe Motor and Intellectual Disabilities: A Retrospective Case Series

**DOI:** 10.7759/cureus.103710

**Published:** 2026-02-16

**Authors:** Shohei Tobu, Yukako Yamaguchi, Minika Yukimoto, Akihiro Maeda, Shuhei Kusano, Yuka Kakinoki, Hiroaki Kakinoki, Maki Kawasaki, Mitsuru Noguchi

**Affiliations:** 1 Department of Urology, Faculty of Medicine, Saga University, Saga, JPN

**Keywords:** blocksom vesicostomy, neurogenic bladder, retrospective study, severe motor and intellectual disabilities, urinary diversion

## Abstract

Background: Urinary tract management in adult patients with severe motor and intellectual disabilities is often challenging because long-term catheter-based management is associated with recurrent urinary tract infections (UTIs), urinary stone formation, catheter-related complications, and increased caregiver burden. Evidence regarding catheter-free urinary management strategies in this population remains limited.

Methods: We retrospectively reviewed six adult patients with severe motor and intellectual disabilities who underwent Blocksom-type cutaneous vesicostomy after failure of conventional urinary management, including indwelling urethral catheters, suprapubic cystostomy catheters, or assisted catheterization. Clinical backgrounds, indications for surgery, postoperative courses, and outcomes were evaluated.

Results: All patients had underlying neurogenic bladder and had experienced recurrent UTIs, urinary stone formation, or difficulty in catheter management prior to surgery. After cutaneous vesicostomy, continuous low-pressure urinary drainage was achieved without the need for indwelling catheters. During postoperative follow-up, no recurrence of febrile UTIs was observed, and urinary tract management stabilized in all cases. In addition, simplification of urinary care contributed to a reduction in caregiver burden and improved living environments.

Conclusions: Blocksom-type cutaneous vesicostomy may be a feasible and effective catheter-free urinary management option for selected adult patients with severe motor and intellectual disabilities who experience refractory complications with conventional catheter-based management. Careful patient selection and shared decision-making are essential when considering this procedure.

## Introduction

In adult patients with severe motor and intellectual disabilities who are unable to void spontaneously, indwelling urethral catheterization or suprapubic cystostomy catheterization is frequently employed as a method of urinary tract management. In clinical practice, these approaches are often chosen in cases where repeated catheterization or continence management is difficult, particularly from a caregiving perspective [1,2]. In many cases, because patients have limited voluntary movement, the physical discomfort associated with catheter placement may not be readily apparent in the short term.

However, long-term management with an indwelling urethral catheter is associated with several complications, including urethral injury during catheter replacement, persistent urethral discomfort, and pain. Consequently, among healthcare professionals with sufficient experience, there is a shared understanding that suprapubic catheterization is preferable to urethral catheterization when long-term urinary drainage is required. In actual urological practice, many clinicians manage patients based on this principle.

Suprapubic cystostomy catheterization offers the advantage of avoiding urethral irritation and, except for the risk of accidental self-removal, generally provides relatively stable urinary drainage in the short term. Nevertheless, long-term indwelling catheterization in the bladder has been reported to result in a wide range of complications, including chronic urinary tract infections (UTIs), recurrent febrile UTIs, formation of renal and bladder calculi, and progressive reduction in bladder capacity or development of a contracted bladder [2,3]. Once such complications occur, their management can be challenging in real-world clinical settings.

Although prolonged indwelling catheterization can cause management difficulties even in the general elderly population, the situation is considerably more complex in patients with severe motor and intellectual disabilities. In Japan, the medical and caregiving systems for adult patients with severe disabilities are considered to have several challenges, particularly in long-term urinary management. Factors such as the aging of family caregivers, declining caregiving capacity, and insufficient coordination between home-based and institutional care may result in catheter-related problems being managed without definitive solutions [4]. As a result, patients with complex and refractory urinary tract management issues tend to accumulate at a limited number of specialized medical institutions, creating a structural burden on healthcare resources.

Against this background, we have actively introduced Blocksom-type cutaneous vesicostomy, first described by Blocksom in 1957 [5], as an alternative urinary management option for adult patients with severe motor and intellectual disabilities who are unable to void spontaneously, in place of conventional urethral or suprapubic catheterization. Cutaneous vesicostomy enables catheter-free urinary management and may suppress UTIs and urinary stone formation by allowing continuous, low-pressure urinary drainage. Furthermore, simplification of urinary care has the potential to reduce caregiver burden and stabilize the patient’s daily living environment.

Previous studies have demonstrated the feasibility and long-term safety of Blocksom-type cutaneous vesicostomy in elderly patients with chronic urinary retention [6,7]. In contrast, this procedure has traditionally been positioned as a urinary diversion primarily for pediatric patients, and reports in adult populations remain limited [8]. To date, there have been no studies focusing on adult patients with severe motor and intellectual disabilities, nor have there been reports evaluating multiple cases or long-term outcomes in this unique population.

Therefore, in this study, we retrospectively analyzed six adult patients with severe motor and intellectual disabilities who underwent cutaneous vesicostomy, focusing on their clinical backgrounds, postoperative courses, and outcomes, with the aim of clarifying the usefulness and limitations of this procedure in this underserved patient population.

The primary objective of this study was to evaluate postoperative urinary tract outcomes, including febrile UTIs, urinary stone recurrence, and upper urinary tract status, in adult patients with severe motor and intellectual disabilities who underwent Blocksom-type cutaneous vesicostomy.

The secondary objectives were to assess procedural safety and changes in urinary management complexity, including caregiver burden. Caregiver burden was assessed qualitatively based on clinical observations and caregiver reports.

This study was designed as a descriptive, hypothesis-generating retrospective case series.

## Materials and methods

This retrospective study was reviewed and approved by the Ethics Committee of Saga University Hospital (Approval No. 2024-02-R-03). According to institutional regulations, the requirement for written informed consent was waived due to the retrospective nature of the study. All patient data were anonymized prior to analysis. This study was conducted as a retrospective case series.

This retrospective case series included adult patients with severe motor and intellectual disabilities who were unable to void spontaneously and underwent Blocksom-type cutaneous vesicostomy for urinary management between January 2015 and December 2023 at Saga University Hospital.

**Figure 1:**
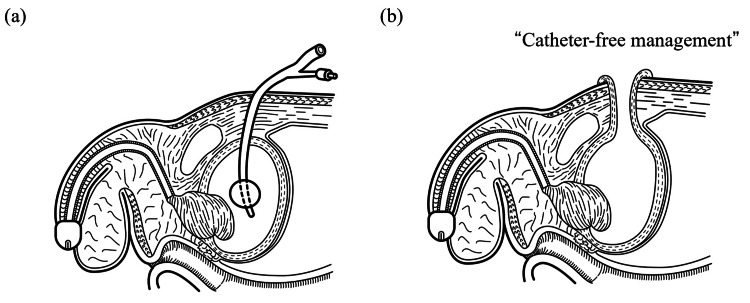
Schematic illustration of urinary diversion techniques. (a) Conventional catheter-based suprapubic cystostomy. (b) Blocksom-type cutaneous vesicostomy. This illustration was independently redrawn for the present study based on the same surgical concept described in our previous publication [7].

Figure 1 illustrates the surgical concept and configuration of the Blocksom-type cutaneous vesicostomy.

## Results

Six adult patients with severe motor and intellectual disabilities underwent cutaneous vesicostomy during the study period. Table 1 summarizes the demographic and clinical characteristics of the six adult patients included in this study.

**Table 1:** Clinical characteristics of adult patients with severe motor and intellectual disabilities who underwent Blocksom-type cutaneous vesicostomy. LTCH, long-term care hospital.

No.	Sex	Age at surgery	Follow-up duration (months)	Place of residence	Reason for vesicostomy	Underlying disease	Height (m)	Weight (kg)	BMI (kg/m^2^)	Procedure-related complications
1	F	38	67	LTCH	Recurrent febrile urinary tract infection associated with long-term urethral catheterization	Cerebral palsy	1.46	33	15.4	None
2	F	42	64	LTCH	Recurrent febrile urinary tract infection associated with long-term urethral catheterization	Cerebral palsy	1.4	39.3	20.1	None
3	M	21	42	LTCH	Recurrent febrile urinary tract infection associated with long-term urethral catheterization	Muscular dystrophy	1.24	26.2	17	None
4	M	35	38	Home	Febrile urinary tract infection and nephrolithiasis complicated by urethral stricture	Muscular dystrophy	1.5	26.5	11.8	None
5	M	64	14	LTCH	Recurrent febrile urinary tract infection associated with long-term urethral catheterization	Cerebral palsy	1.48	32.1	14.8	None
6	M	43	13	LTCH	Recurrent febrile urinary tract infection associated with long-term urethral catheterization	Cerebral palsy	1.45	42.3	20.1	None

Case 1

A 43-year-old female with severe motor and intellectual disabilities was admitted to a residential care facility. She had exhibited developmental delay since infancy and had a history of cerebral palsy. Neurogenic bladder had been diagnosed in early childhood, and she underwent evaluation following recurrent UTIs. Voiding cystourethrography revealed bilateral vesicoureteral reflux (VUR) with associated bladder diverticula.

Assisted clean intermittent catheterization (five times daily) and anticholinergic therapy were initiated; however, febrile UTIs continued to recur. At the age of 37 years, she developed sepsis secondary to an upper UTI and required intensive care management. Imaging studies demonstrated progression of VUR, and infection control remained difficult despite pharmacological treatment and prophylactic antibiotic therapy.

**Figure 2:**
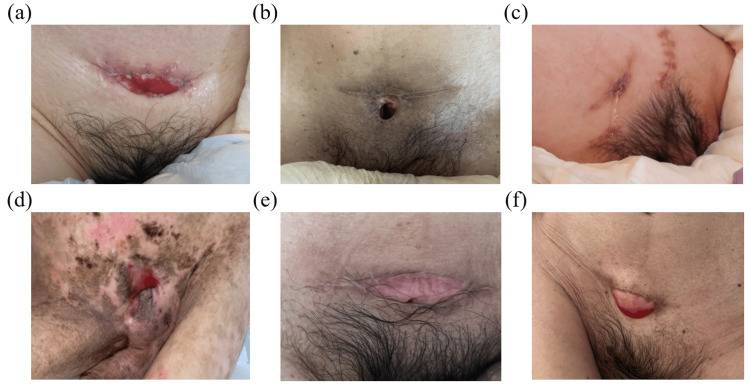
Postoperative appearance of cutaneous vesicostomy in six adult patients with severe motor and intellectual disabilities. (a) Case 1: Five years after surgery. (b) Case 2: Five years after surgery. (c) Case 3: Three years after surgery. (d) Case 4: More than three years after surgery. (e) Case 5: More than one year after surgery. (f) Case 6: More than one year after surgery.

Because recurrent febrile UTIs significantly affected her general condition and increased caregiver burden, definitive surgical intervention was considered. At 38 years of age, bilateral ureteral reimplantation was performed in combination with cutaneous vesicostomy. Postoperatively, urinary drainage from the vesicostomy was satisfactory, and hydronephrosis improved to a mild degree. Five years after surgery, no recurrence of febrile UTI has been observed, and both her general condition and urinary management remain stable. The current appearance of the cutaneous vesicostomy is shown in Figure 2A.

Case 2

A 47-year-old female with severe motor and intellectual disabilities associated with microcephaly and epilepsy had been residing in a long-term care facility. She had experienced recurrent UTIs since adolescence and underwent suprapubic cystostomy at the age of 27 years.

Subsequently, she developed recurrent bladder stones and underwent cystolithotomy at 35 years of age. From approximately 42 years of age, accidental self-removal of the indwelling bladder catheter occurred frequently, and computed tomography revealed multiple bladder stones. Conservative management failed to control stone formation and infection, and caregiver burden increased.

After discussion with her family and care facility, cutaneous vesicostomy was planned to simplify urinary management and was performed at 42 years of age. Postoperatively, she experienced transient low-grade fever, which resolved promptly with antibiotic treatment. Renal ultrasonography showed no hydronephrosis, and she was discharged in stable condition. At five years after surgery, no recurrence of bladder stones or worsening of UTIs has been observed, and urinary management remains stable with a reduced caregiver burden. The appearance of the cutaneous vesicostomy at five years postoperatively is shown in Figure 2B.

Case 3

A 24-year-old male with myotonic dystrophy had undergone tracheostomy in early childhood and gastrostomy during adolescence and was living in a residential care facility. Neurogenic bladder had been managed with clean intermittent catheterization; however, with growth, he developed recurrent urinary tract calculi, including multiple renal and bladder stones.

The calculi were infection-related, and urinary findings showed poor improvement despite antibiotic therapy. Imaging studies demonstrated bilateral renal atrophy. Given the increasing complexity of urinary management and difficulty in infection control, surgical intervention was considered to achieve low-pressure urinary drainage and prevent further infections.

At 21 years of age, cystoscopic evaluation and cutaneous vesicostomy were performed. Postoperatively, urinary drainage through the vesicostomy was satisfactory without exacerbation of UTIs. The frequency of UTIs and stone-related symptoms decreased thereafter, resulting in long-term stabilization of urinary tract management. The appearance of the cutaneous vesicostomy at three years postoperatively is shown in Figure 2C.

Case 4

A 38-year-old male with congenital muscular dystrophy and severe motor and intellectual disabilities was receiving home-based care with tracheostomy and gastrostomy. He had a neurogenic bladder with voiding dysfunction and had required assisted catheterization since 22 years of age. Due to urethral stricture causing catheterization difficulty, a suprapubic cystostomy catheter was placed at 26 years of age.

From approximately 33 years of age, he developed recurrent pyelonephritis and urinary tract stones and experienced repeated febrile episodes despite symptomatic treatment with antibiotics. Because conservative management failed to control infections, cutaneous vesicostomy was performed at 35 years of age.

After surgery, the frequency of UTIs decreased, and his general condition stabilized. Urinary management was simplified, enabling continuation of home-based care. The appearance of the cutaneous vesicostomy more than three years postoperatively is shown in Figure 2D.

Case 5

A 65-year-old male with cerebral palsy had been managed with a long-term indwelling urethral catheter. He developed progressive bladder stone enlargement and recurrent UTIs. At 64 years of age, cystolithotomy combined with cutaneous vesicostomy was performed as definitive treatment.

Postoperatively, his clinical course was favorable, and UTIs resolved completely. No recurrence of urinary stones has been observed, and simplification of urinary management led to improvement in the caregiving environment. The appearance of the cutaneous vesicostomy more than one year after surgery is shown in Figure 2E.

Case 6

A 44-year-old man with cerebral palsy had been managed with a long-term indwelling urethral catheter. He was bedridden and had a chronic neurogenic bladder with recurrent bladder stones and UTIs, which had been treated conservatively with antibiotics alone.

At 43 years of age, a cutaneous vesicostomy was performed. Postoperatively, urinary drainage stabilized, and signs of infection resolved. No recurrence of urinary tract complications has been observed, and both caregiver burden and the patient’s general condition improved. The appearance of the cutaneous vesicostomy more than one year after surgery is shown in Figure 2F.

All procedures were completed successfully without intraoperative complications. No perioperative mortality was observed. One patient experienced a transient postoperative low-grade fever, which resolved promptly with conservative antibiotic treatment.

During the follow-up period, urinary drainage through the cutaneous vesicostomy was satisfactory in all patients. Recurrent febrile UTIs decreased markedly after surgery, and no patient experienced worsening of upper urinary tract dilation. No recurrence of bladder stones was observed in patients who had undergone prior cystolithotomy.

Overall, urinary management was simplified following cutaneous vesicostomy, resulting in stable clinical courses and a reduction in caregiver burden throughout the follow-up period.

## Discussion

In this retrospective case series, we evaluated the usefulness and limitations of Blocksom-type cutaneous vesicostomy in six adult patients with severe motor and intellectual disabilities who were unable to void spontaneously and had experienced substantial difficulty with conventional catheter-based urinary management. All patients had underlying neurogenic bladder and had previously been managed with indwelling urethral catheters, suprapubic cystostomy catheters, or assisted catheterization at referring institutions. However, they were repeatedly affected by chronic refractory UTIs, recurrent febrile UTIs, renal and bladder stone formation, catheter obstruction, and accidental catheter removal, resulting in long-term failure of urinary tract management and subsequent referral to our institution. After cutaneous vesicostomy, none of the patients experienced recurrence of febrile UTIs during the postoperative follow-up period.

Urinary tract management in adults with severe motor and intellectual disabilities is constrained by multiple factors, including activities of daily living, caregiving capacity, access to medical care, and comorbid conditions such as respiratory dependence, gastrostomy, seizure disorders, and severe scoliosis. In particular, indwelling urethral catheters often require frequent replacement and are prone to obstruction or accidental self-removal, leading to repeated medical visits and difficulty in infection control. Similarly, long-term suprapubic catheterization is associated with biofilm formation and acts as a nidus for urinary stone development, further complicating long-term management [9-11].

In addition, patients with severe motor and intellectual disabilities often have limited ability to express symptoms, which can delay the detection of infection. This delay increases the risk of progression to sepsis or deterioration of renal function. Long-term catheter-based urinary management is well known to be associated with recurrent UTIs, biofilm formation, infection-related calculi, and sepsis, particularly in patients with neurogenic bladder dysfunction [2,8,12].

The advantages of cutaneous vesicostomy can be summarized in three key points: (1) continuous, low-pressure urinary drainage that suppresses elevations in intravesical pressure and may contribute to upper urinary tract protection; (2) avoidance of indwelling foreign bodies such as catheters, thereby reducing catheter-related complications, including obstruction, accidental removal, and frequent replacement; and (3) simplification of urinary management, which may reduce the burden on families and institutional caregivers.

In patients with neurogenic bladder, maintenance of low-pressure urinary drainage and protection of the upper urinary tract are critical determinants of long-term prognosis, as reducing detrusor storage pressure and preserving upper tract function are key treatment goals in neurogenic lower urinary tract dysfunction [12-14].

Previous studies in patients with spinal cord injury have demonstrated that appropriate urinary management contributes to preservation of renal function and maintenance of quality of life [15,16].

In the present series, postoperative improvement of hydronephrosis and suppression of recurrent UTIs were observed in several patients, supporting the clinical usefulness of this procedure in stabilizing urinary tract management. Furthermore, in patients with recurrent urinary stones, combining stone treatment with cutaneous vesicostomy may have contributed to the prevention of recurrence and further simplification of long-term care.

Nevertheless, careful consideration of indications is required when selecting this procedure. Cutaneous vesicostomy necessitates ongoing care of the cutaneous stoma and may be associated with local complications such as skin irritation due to urine exposure, infection, or stomal stenosis. In addition, surgical invasiveness and anesthesia-related risks are not negligible in patients with severe motor and intellectual disabilities who often have compromised respiratory function, poor nutritional status, or poorly controlled seizures. In our cohort, the severity of underlying conditions influenced overall outcomes, underscoring the reality that improvement in urinary tract management alone does not necessarily determine prognosis. Therefore, this procedure should be selected with clearly defined goals, namely, suppression of urinary tract complications and reduction of caregiving burden, and with shared decision-making involving the patient (when possible), family members, care facilities, and a multidisciplinary healthcare team.

The significance of this study lies in presenting a series of adult patients with severe motor and intellectual disabilities who underwent cutaneous vesicostomy, thereby highlighting both the practical challenges of urinary tract management in real-world clinical settings and the potential of a catheter-free management strategy. In adult patients with severe disabilities, continuation of urethral or suprapubic catheterization has traditionally been regarded as inevitable, and definitive interventions are often not pursued even after complications become apparent. Our experience suggests that urinary tract management in this population can be reconsidered not only as a means of maintenance but also as an opportunity to reduce complications, decrease medical dependency, and improve the living environment.

This study has several limitations. It was a single-center, retrospective analysis involving a small number of patients, and follow-up duration, as well as evaluation of outcomes, such as UTI frequency before and after surgery, antibiotic usage, stone recurrence, renal function changes, and quantitative assessment of caregiver burden, were not fully standardized. In addition, the absence of a control group receiving continued urethral or suprapubic catheterization limits rigorous assessment of causality. Future studies should incorporate standardized indicators, including infection-related events and healthcare utilization (emergency visits, hospitalizations, catheter exchanges), as well as comprehensive evaluations of patient, family, and caregiver quality of life, to further clarify appropriate indications and long-term outcomes of this procedure.

## Conclusions

Cutaneous vesicostomy provided safe and stable urinary drainage in adult patients with severe motor and intellectual disabilities who experienced difficulty with conventional urinary management. In this retrospective case series, the procedure was associated with a reduction in recurrent UTIs, stabilization of the upper urinary tract, and simplification of daily urinary care, resulting in reduced caregiver burden. Cutaneous vesicostomy may represent a useful alternative urinary diversion option for selected adult patients in whom long-term catheter-based management is problematic.
